# Supplementary material for: Sympathetic Innervation Modulates Mucosal Immune Homeostasis and Epithelial Host Defense
Source: Cells. 2022 Aug 21;11(16):2606. doi: 10.3390/cells11162606 (PMC9406312; doi:10.3390/cells11162606)
Supplement: Supplementary file 1 [file cells-11-02606-s001.zip › cells-1741455-supplementary/Cells-1741455_Supplementary_figures.pdf]

Supplementary Figures

Figure S1

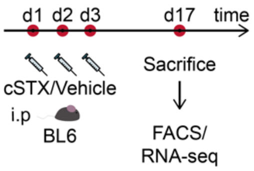

Figure S1: The experimental setup for 6-OHDA and vehicle treatment.

Figure S2

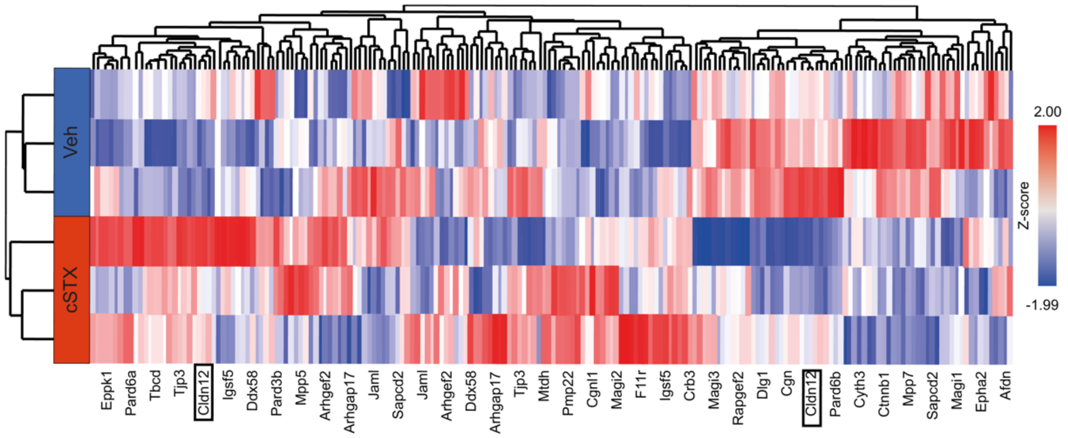

Figure S2: The heatmap analysis showing transcriptional differences in genes related to tight junction in cSTX compared to vehicle- treated mice

Figure S3A

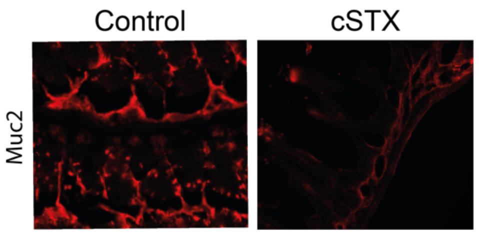

Figure S3B

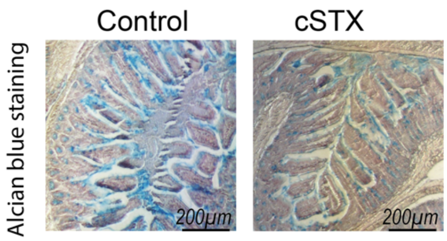

Figure S3A: Diminished Muc2 protein expression in cSTX compared to control mice (A).
Figure S3B: Reduced numbers of alcian blue positive goblet cells in cSTX compared to control mice (B).

Figure S4A

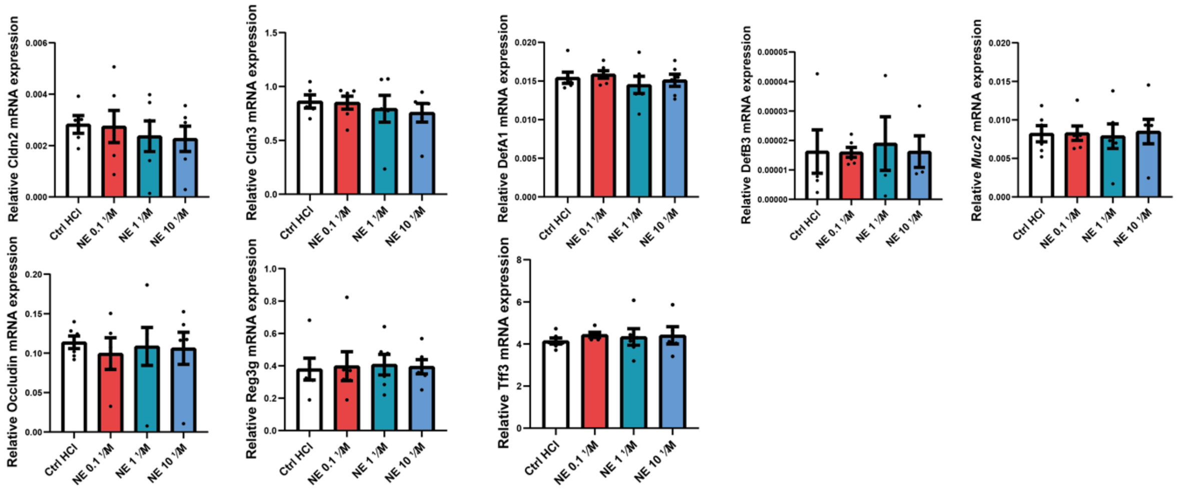

Figure S4A: mRNA levels of Cldn2, Cldn3, Defa1, DefB3, Muc2, Ocln, Reg3g, and Tff3 showing no changes after 72 hours of stimulation with on norepinephrine stimulated organoids, compared with the vehicle-stimulated organoids.

Figure S4B

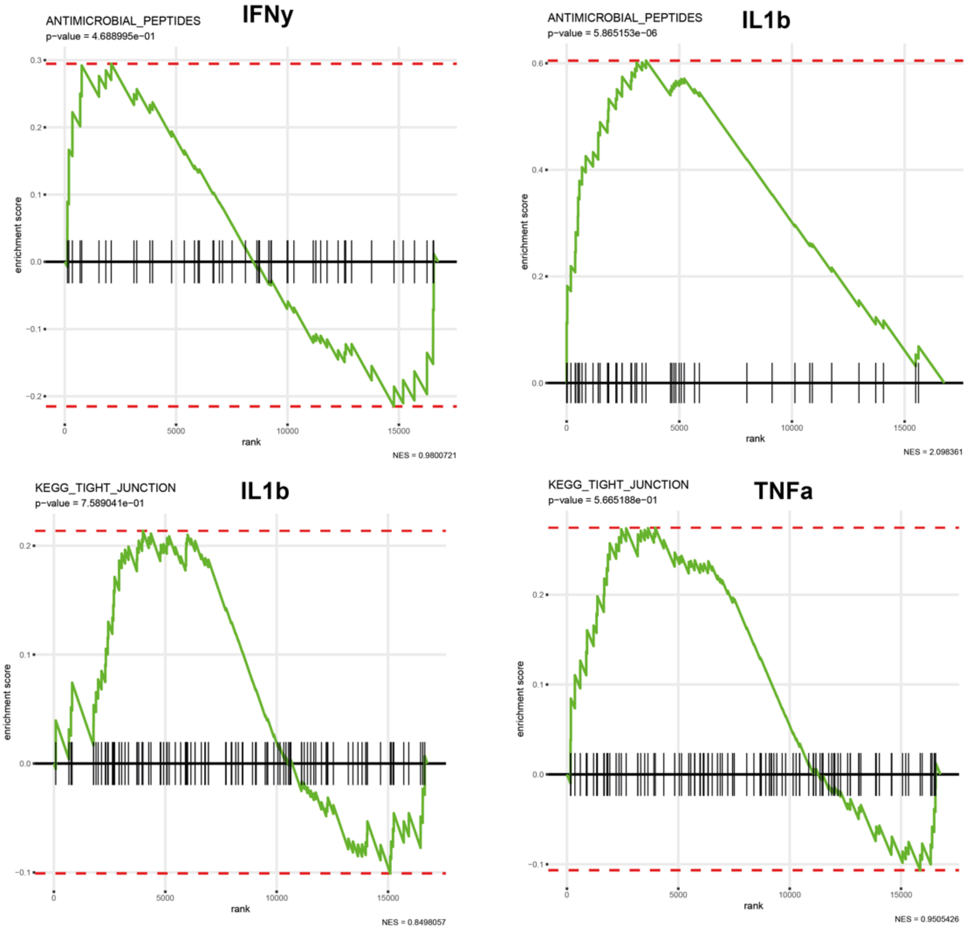

Figure S4B: Gene set enrichment analyses (GSEA) showing enrichment of genes expressing tight junctions following treatment with IFN-y, IL1b and antimicrobial peptides upon treatment with IL1b and TNF-a

Table S1: Primer sequences used for qPCR analysis.

	Forward	Reverse
Arg1	TTGGGTGGATGCTCACACTG	TTGCCCATGCAGATTCCC
CD163	GTGCTGGATCTCCTGGTTGT	CGTTAGTGACAGCAGAGGCA
IL6	AAGTCGGAGGCTTAATTACACATGT	CCATTGCACAACTCTTTTCTCATT
IL1b	TTGACGGACCCCAAAAGATG	CAGGACAGCCCAGGTCAAAG
TNFa	CTGAACTTCGGGGTGATCGG	GGCTTGTCACTCGAATTTTGAGA
Muc2	TGCCCAGAGAGTTTGGAGAGG	CCTCACATGTGGTCTGGTTG
TFF3	CTCTGTCACATCGGAGCAGTGT	TGAAGCACCAGGGCACATT
Cldn2	CCACAAGCAGGCTCAAGAAG	TTCGCCTTTCTCTGGACCTA
Cldn3	GCAAGCAGACTGTGTGTCGT	TACCGTCACCACTACCAGCA
Ocln	CATAGTCAGATGGGGGTGGA	ATTTATGATGAACAGCCCCC
Defa1	CAGGCCGTATCTGTCTCCTT	ATGACCCTTTCTGCAGGTTC
Reg3g	TTCCTGTCCTCCATGATCAAAA	CATCCACCTCTGTTGGGTTCA
ß3 defensin	GTTTGCATTTCTCCTGGTGC	GCCTCCTTTCCTCAAACAACT
Lyz1	GAGACCGAAGCACCGACTATG	CGGTTTTGACATTGTGTTCGC
GAPDH	GGGAAGCCCATCACCATCTT	GCCTCACCCCATTTGATGTT
