# Supplementary figures and images for: Sympathetic Innervation Modulates Mucosal Immune Homeostasis and Epithelial Host Defense
Source: Cells. 2022 Aug 21;11(16):2606. doi: 10.3390/cells11162606 (PMC9406312; doi:10.3390/cells11162606)

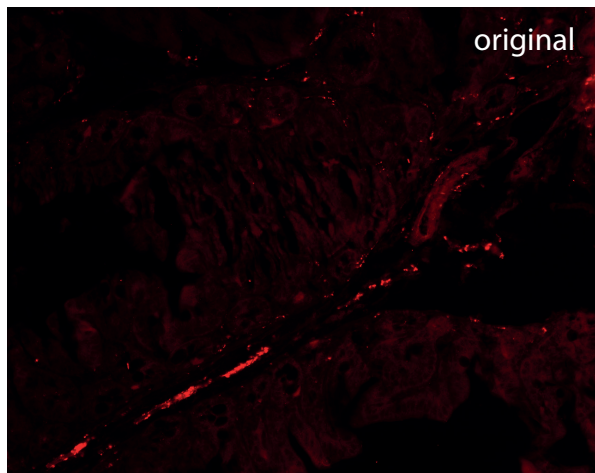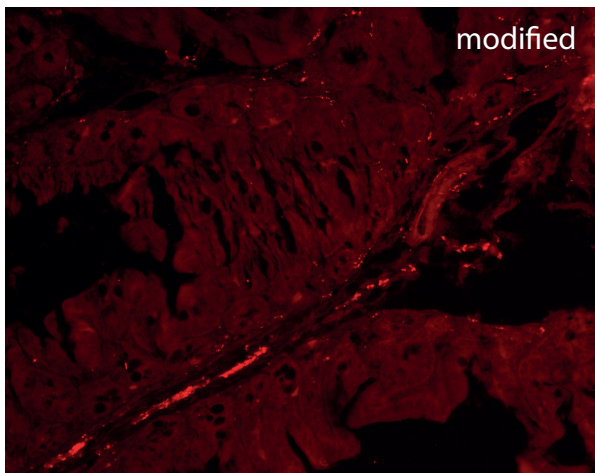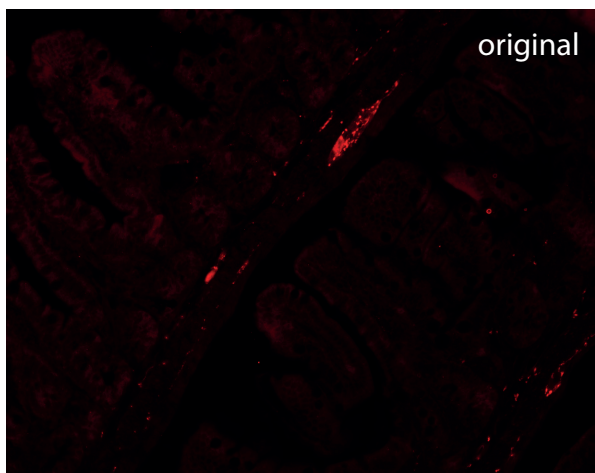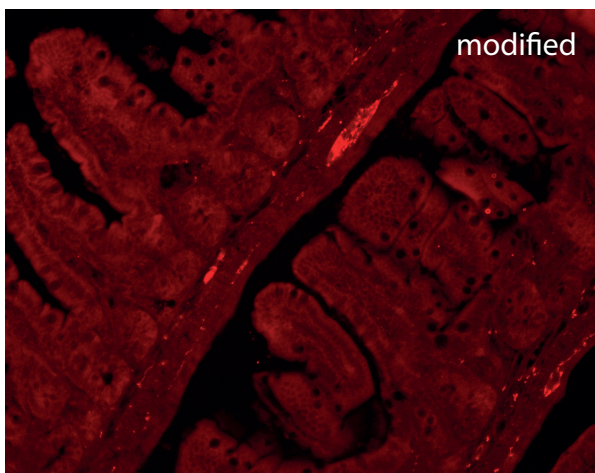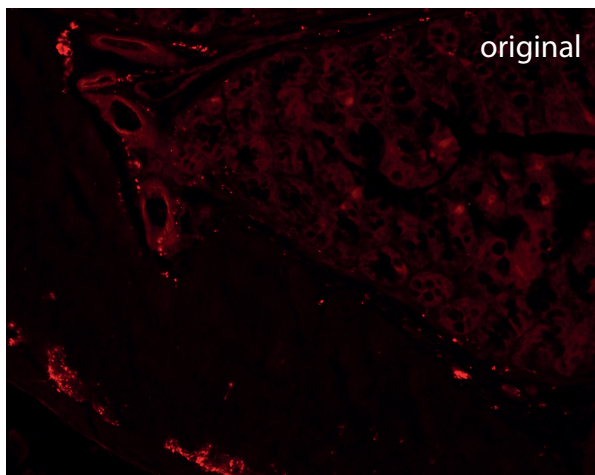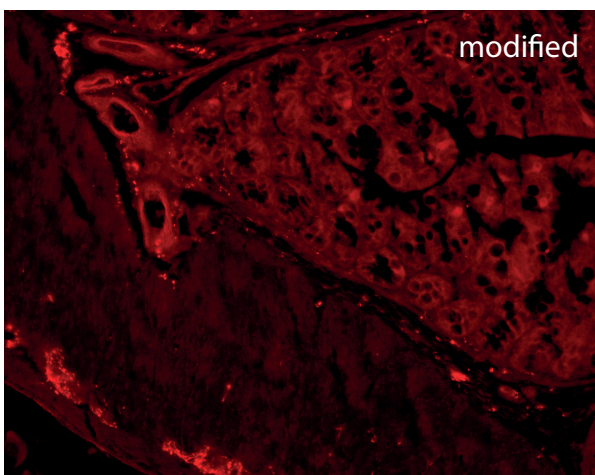

Supplement: Supplementary file 1 [file cells-11-02606-s001.zip › cells-1741455-supplementary/File S1-Enlarged figures from Figure 1A-C/comparative TH images.pdf]
